# Childhood Vaccinations and Type 1 Diabetes

**DOI:** 10.3389/fimmu.2021.667889

**Published:** 2021-08-26

**Authors:** Susanna Esposito, Elena Mariotti Zani, Lisa Torelli, Sara Scavone, Maddalena Petraroli, Viviana Patianna, Barbara Predieri, Lorenzo Iughetti, Nicola Principi

**Affiliations:** ^1^Paediatric Clinic, Department of Medicine and Surgery, University Hospital, University of Parma, Parma, Italy; ^2^Pediatric Unit, Department of Medical and Surgical Sciences of the Mothers, Children and Adults, University of Modena and Reggio Emilia, Modena, Italy

**Keywords:** autoimmunity, infection, type 1 diabetes, vaccination, vaccine

## Abstract

Type 1 diabetes (T1D) is the most common paediatric endocrine disease, and its frequency has been found to increase worldwide. Similar to all conditions associated with poorly regulated glucose metabolism, T1D carries an increased risk of infection. Consequently, careful compliance by T1D children with schedules officially approved for child immunization is strongly recommended. However, because patients with T1D show persistent and profound limitations in immune function, vaccines may evoke a less efficient immune response, with corresponding lower protection. Moreover, T1D is an autoimmune condition that develops in genetically susceptible individuals and some data regarding T1D triggering factors appear to indicate that infections, mainly those due to viruses, play a major role. Accordingly, the use of viral live attenuated vaccines is being debated. In this narrative review, we discussed the most effective and safe use of vaccines in patients at risk of or with overt T1D. Literature analysis showed that several problems related to the use of vaccines in children with T1D have not been completely resolved. There are few studies regarding the immunogenicity and efficacy of vaccines in T1D children, and the need for different immunization schedules has not been precisely established. Fortunately, the previous presumed relationship between vaccine administration and T1D appears to have been debunked, though some doubts regarding rotavirus vaccines remain. Further studies are needed to completely resolve the problems related to vaccine administration in T1D patients. In the meantime, the use of vaccines remains extensively recommended in children with this disease.

## Background

The anti-infective vaccines included in the immunization schedule adopted by most countries for protecting children can cause several problems when these prophylactic measures have to be given to subjects at risk of or with overt type 1 diabetes (T1D). T1D is the most common paediatric endocrine disease, and its frequency has been found to increase worldwide with relevant medical, social and economic issues (1). According to the International Diabetes Federation, it was estimated that more than 1.1 million children and adolescents around the world were living with T1D in 2019 compared to 860,000 in 2013 (2). Similar to all conditions associated with poorly regulated glucose metabolism and persistent hyperglycaemia, including type 2 diabetes (T2D), T1D carries an increased risk of infection. Consequently, careful compliance by T1D children with schedules officially approved for child immunization by national governments is strongly recommended by scientific societies (3).

A list of vaccines for the prevention of the most common infectious diseases diagnosed in adults with T1D and T2D is recommended for children with T1D (4). However, because patients with T1D show persistent and profound limitations in immune function (5, 6), vaccines may evoke a less efficient immune response, with corresponding lower protection. A larger use of boosters to maintain elevated protection has been suggested for some vaccines (3). Moreover, T1D is an autoimmune condition (7–11) that develops in genetically susceptible individuals (12), when epigenetic or environmental factors act as triggers and modulate the penetrance of susceptibility genes (13). For example, associations of T1D development with some nutrients such as cow’s milk and gluten as well as increased maternal age and rate of postnatal growth, vitamin D deficiency, chemical exposure, and gut dysbiosis have been suggested (14–18). Nevertheless, most data regarding T1D triggering factors appear to indicate that infections, mainly those due to viruses, play a major role. Enteroviruses and herpesviruses have frequently been associated with T1D development, but other viruses, including some of those used to prepare vaccines such as rotavirus, influenza viruses, rubella and mumps viruses, have also been found to cause pancreatic infection and autoimmunity (19). Accordingly, the use of vaccines is being debated, as this hypothesis is reinforced by some epidemiological evidence (20–22). In this narrative review, these issues are discussed to define the most effective and safe use of vaccines in patients at risk of or with overt T1D.

## Infections in Type 1 Diabetes Patients

### Risk of Infection

Together with multisystem microangiopathy and macrovascular disease (23), immune compromise is the most common complication of poor glycaemic control. The immune response is disrupted in any type of diabetes, and both the innate and adaptive immune systems are impaired. Defects in pathogen recognition, suppression of cytokine production, poor neutrophil and macrophage recruitment and function, alteration in natural killer cell activity, and inhibition of antibodies and complement effectors have been repeatedly reported in both experimental animals and humans with T1D and T2D (24). Indeed, despite a few exceptions (25, 26), studies involving adults have clearly shown that patients with T1D are at increased risk of infection (27–29). In a 12-month prospective cohort study carried out in the Netherlands from May 2000 through April 2002, it was demonstrated that the incidence of lower respiratory tract infections was significantly higher among 705 T1D patients than among 18,911 controls (adjusted odds ratio [AOR], 1.42; 95% confidence interval [CI] 0.96–2.08), with urinary tract infection (AOR, 1.96 [95% CI 1.49–2.58]), bacterial skin and mucous membrane infections (AOR, 1.59; 95% CI 1.12–2.24) and mycotic skin and mucous membrane infection (AOR, 1.34; 95% CI 0.97–1.84) being common (30).

Furthermore, risk increased with recurrence. These findings were confirmed by a more recent study performed using English primary care data collected during 2010-2015 in which the incidence and outcome of infections were related to the degree of T1D severity measured through HbA1c evaluation (31). In this study, 5,863 T1D patients were matched with 8,231 controls, and patients requiring a prescription or hospitalization or who died were compared. The results showed that long-term infection risk rose with increasing HbA1c for most outcomes. Hospitalization for infection was significantly more frequent in patients with T1D than in controls (incidence rate ratio [IRR] 3.34; 95% CI 2.82–3.96), and poor glycaemic control was associated with an increased risk: subjects with HbA1c ≥11% had an IRR of 8.47 (95% CI 5.86-12.24), whereas those with optimal control had an IRR of 1.41 (95% CI 1.36–1.47). The largest relative associations between the poorest level of glycaemic control and optimal control were seen for bone and joint infections, endocarditis, and sepsis. In addition, a strict relationship between poor glycaemic control and infection severity has been recently shown in severe acute respiratory syndrome coronavirus 2 (SARS-CoV-2)-infected patients. T2D was found to be among the most common medical condition in adult patients developing COVID-19 (32), and it was associated with an almost fourfold greater risk for severe disease and death (OR 3.68, 95% CI 2.68–5.03; P < 0.001) (33).

Unfortunately, the incidence of infections in T1D paediatric patients has been poorly studied. Most of the available evidence comes from studies conducted in adults and the evidence suggesting a higher risk of infections in children with T1D is extremely weak. On the other hand, it is common experience of clinicians looking after these patients that they do not show any increased risk of infections, especially severe infections. Nonetheless, as children with poor glycaemic control have immune and metabolic disorders similar to those found in adults, it seems likely that children may have a risk of infection substantially similar to that in adults. Support for this hypothesis can be found in a retrospective study in the USA using data collected from 2008 to 2014 at 44 freestanding children’s hospitals across the country (34). The authors analysed the clinical characteristics of children and adolescents with T1D who presented to the emergency department (ED) or were hospitalized for infection management. A total of 104,739 cases were studied: 34,332 visited the ED, and 60,407 visited the hospital. The data showed that medical attention for infections is routinely given to paediatric patients with T1D and that the need for assistance for these patients increases over time in parallel with the increase in T1D cases, with a relevant impact on assistance costs that increased from $189 to $218 million dollars per year. Considering COVID-19, overall, the accumulating evidence suggests that children with T1D infected with severe acute respiratory syndrome coronavirus 2 (SARS-CoV-2) have similar disease outcomes as peers without diabetes (35, 36).

### Type of Infections in Patients With Type 1 Diabetes (T1D)

As previously highlighted, some infections are common in T1D patients. Respiratory infections caused by *Mycobacterium tuberculosis, Staphylococcus aureus*, gram-negative bacteria and fungi may occur with an increased frequency (37, 38). Infections due to *Streptococcus pneumoniae* (39) and influenza viruses deserve particular attention because they are extremely more common than in healthy subjects and are associated with a significantly increased risk of hospitalization and death (40, 41). Skin and soft tissue infections ranging from folliculitis, furunculosis, and subcutaneous abscesses to necrotizing fasciitis are frequently caused by methicillin‐resistant *S. aureus* and *Staphylococcus epidermidis* (42). Urinary tract infections are described in diabetic subjects up to 10 times more frequently than in healthy subjects. Moreover, these infections are 4 times more commonly associated with bacteraemia than in healthy subjects and are frequently due to multi‐drug‐resistant microbes (43). Among gastrointestinal diseases, hepatitis B (HB) (44), hepatitis C (45) and oral and oesophageal candidiasis (46) are common in those with T1D.

## Immune Response and Clinical Efficacy of Recommended Vaccines in Patients with Type 1 Diabetes (T1D)

Children and adolescents with T1D are considered a special population requiring vaccination according to the immunization schedule recommended for healthy subjects, with particular attention to pneumococcal and influenza vaccines. Boosters of pneumococcal vaccines may be necessary, and influenza vaccines must be rigorously administered each year (3). In adults with T1D, there are specific recommendations for the administration of the hepatitis B (HB) vaccine, tetanus/diphtheria/acellular pertussis vaccine, pneumococcal vaccine, influenza vaccine and herpes zoster vaccine according to age and previous immunizations (4).

However, despite the well-known impairment of immune system function in T1D patients, the immune response to commonly recommended vaccines in these subjects has been poorly studied. Moreover, the results of the few available studies are conflicting and do not allow us to draw definitive conclusions regarding the real protection offered by the different vaccines in a single T1D patient. Evaluation of the immune response of 20 T1D patients to hepatitis A (HA), diphtheria and pneumococcal polysaccharide vaccines showed that T1D patients had a significantly impaired primary antibody response to HA vaccine (P = 0.017) and diphtheria toxoid (P = 0.004) compared to healthy controls but that the response to pneumococcal polysaccharide was normal (47). In another study enrolling 36 children with T1D and a similar number of age-matched healthy controls in which the immune response to conjugate pneumococcal vaccine, Hib vaccine and tetanus/diphtheria vaccine was compared, no difference in antibody levels against the antigens tested was found between the groups. However, after a booster dose, the median level against pneumococcal serotypes was significantly lower in the T1D patients than in the controls (2.3 g/mL [range, 0.05 to 664.7 g/mL] and 6.1 g/mL [0.12 to 203.36 g/mL]), respectively, suggesting reduced immune memory in the former (48).

Regarding the immune response to the HB vaccine, studies carried out approximately 20 years ago showed that both the immediate and long-term immune responses to the HB vaccine in T1D patients did not differ from those of healthy subjects (49–54). Paradigmatic in this regard are the studies of Marseglia et al., who monitored HBsAb titres immediately after the usual schedule of immunization (0, 1 and 6 months) as well as 4 years later in T1D children/young adults and healthy subjects (4.5 to 27.5 years of age). In both cases, the immune response was similar in the T1D patients and controls. A few weeks after the booster dose (49), 3 (4.6%) of 65 T1D patients and 3 (1.7%) of 174 age- and sex-matched healthy subjects were considered to have a low (HBsAb titre = 10 IU/L) or no (HBsAb titre, < 2 IU/L) response. Moreover, the median HBsAb titre was similar in the responding patients (120 IU/L) and controls (125 IU/L). There were no significant correlations between antibody titre and age, diabetes duration, or HbA1c or insulin requirement. After 4 years, mean anti-HBs log-titres were 1.95 ± 0.88 in T1D patients and 2.18 ± 0.64 in controls (P=0.11). Additionally, the number of subjects with protective antibody concentrations (anti-HBs >10 IU/l) was 50/54 (92%) among T1D patients and 67/70 (96%) among controls (P=0.70) (50). More recent studies have shown the opposite. Leonardi et al. reported significantly more common detection of protective serum anti-HBs antibody levels in previously immunized children among healthy subjects (84%) than patients with diabetes (58.2%) (P < 0.0001), regardless of age or duration and metabolic control of T1D. Moreover, among children with antibodies, the T1D children had significantly lower antibody values (58 ± 112.9 mIU/mL *vs* 266.49 ± 335.85 mIU/mL, respectively; P < 0.0001) (55). Similar results were reported by Elrashidy et al., who found protective anti-HBs levels in only 30.2% of children with the disease compared to 60% of healthy controls (P < 0.001), which was independent of the age of patients and the duration of T1D (56). Finally, by analysing the serological response to HBV vaccine in 69 T1D patients and 79 healthy controls who had received the third dose 6.8 and 4.7 years prior, Zanoni et al. (57) showed that although the total number of subjects with protective antibody levels was quite similar in both groups (72% *vs* 77%, respectively), mean serum anti HBs antibody concentrations were lower in the patients than in the controls (75 ± 149 mIU/mL *vs* 169 ± 268 mUI/mL, respectively; P = 0.0068).

Dissimilar results were also reported when the influenza vaccine was evaluated. A study enrolling 105 T1D subjects aged 9-30 years who were randomized to receive either a virosomal or a standard subunit influenza vaccine showed that serum haemagglutinin inhibition antibody titres against the three viruses included in the vaccines at one month post vaccination met the requirements for immunogenicity, with high seroprotection rates (>95%) for strains A/H1N1 and A/H3N2 and seroprotection of 73% and 70% for the virosomal and subunit vaccine for strain B, respectively (58). Similar results were obtained by the same authors in a further study in which T1D paediatric patients who received an influenza MF59-adjuvanted vaccine were evaluated (59). However, in a previous study, it was found that the incidence of non-response to the H3N2 and influenza B components of a trivalent vaccine was substantially lower in T1D patients than in healthy controls (100% *vs* 78% and 80 *vs* 44%, respectively, p<0.05). Moreover, the delayed-type hypersensitivity reaction to influenza antigen was significantly decreased in patients with worse glycaemic control (P < 0.01) (60).

Overall, the real efficacy of immunization in T1D patients has not been established. As most T1D paediatric patients receive the recommended vaccines at the proper time, the effect of no vaccination cannot be easily evaluated. In fact, in a systematic review and meta-analysis of the effectiveness of influenza vaccines in patients with diabetes published in 2015 (61), no data for children with T1D could be analysed because relevant studies were unavailable. Furthermore, studies carried out in adults generally consider patients with T1D and T2D together, and the importance of T1D in conditioning the efficacy of vaccines has not been evaluated. Regardless, the previously cited systematic review and meta-analysis indicates that most studies to date have a very low quality that makes it impossible to determine to what extent vaccines are effective, even though they suggest some beneficial effects of influenza immunization for patients with diabetes.

## Risk of Type 1 Development After Administration of Vaccines

A number of experimental and clinical observations have suggested a potential relationship between infection and T1D development. In experimental animals, viral infections, particularly those due to coxsackieviruses, may cause pancreatic infections and lead to T1D development. In humans, the association between recurrent respiratory tract infections in the first semester of life and the development of pancreatic islet autoimmunity with overt T1D at approximately 8 years of age have been reported (62, 63). Enterovirus (EV) epidemics have also been associated with an increased incidence of T1D. For instance, evidence of infection and detection of EV in the blood and stool were several times more common in children with T1D than in controls (64–67). Similar, although less stringent, results have been obtained for Epstein-Barr virus (68, 69). Among viruses included in vaccines, influenza (70), rubella (71), mumps (72) and rotavirus (73) were initially considered potential triggers of T1D, though the results of recent studies seem to exclude this risk for influenza (74) as well as mumps and rubella (75). Doubt remains with regard to rotavirus, though a higher incidence of T1D among children with clear evidence of a previous rotavirus infection has been reported (76).

Four mechanisms have been proposed to explain how viruses lead to autoimmunity: molecular mimicry, in which virus proteins bearing similar sequences to pancreatic beta cell components activate autoreactive T cells (77); bystander activation, in which beta-cell proteins released during viral infection are captured by antigen-presenting cells that present host epitopes and activate immune response (78); epitope spreading, in which immune responses to endogenous epitopes secondary to the release of self-antigens during viral induced chronic inflammatory pancreatic disease are the basis of autoimmunity (79); and cryptic antigens, in which cryptic self-determinants are presented to T cells in amounts sufficient to induce autoimmunity (80). For molecular mimicry, this hypothesis has been substantiated by several clear lines of evidence. As an example, potential cross-reactivity between structural components of coxsackievirus and human cytomegalovirus and a pancreatic beta-cell component has been reported (81, 82) as well as between the VP1 protein of enterovirus and the beta-cell antigen tyrosine phosphatase IA-2 (83). There are also similarities between islet antigen-2 (IA-2), an autoantigen associated with T1D, and the VP7 protein of a human G3P rotavirus strain. Moreover, cross-reactivity of T cells generated against rotavirus VP7 peptide with IA-2, and vice versa, has been reported (84).

The hypothesis that vaccines might have the same potential role already reported for some viruses and trigger T1D development was initially strongly substantiated by a number of studies. In most cases, the temporal association between vaccine introduction in the immunization schedule of infants and children and the sudden increase in T1D incidence in the same paediatric population was considered key for demonstrating that vaccines might cause T1D. For example, clustering of T1D cases at approximately 2-4 years after *Haemophilus influenzae* type B (Hib) vaccine, pertussis vaccine, combined measles, mumps, rubella (MMR) vaccine, and BCG vaccine administration has been reported (85). As the time distance between the onset of autoantibodies against pancreatic beta cells and the development of overt T1D is generally the same, this was considered strong evidence of the risk related to vaccine use. A relationship between vaccine and T1D development and the time of the first vaccine administration was proposed. Certain vaccines, such as the HB vaccine and BCG vaccine, might decrease the risk of developing T1D if given at birth; first vaccination at 2 months of life or later might also increase the risk (86). However, most experts did not attribute significant importance to these findings, and recommendations for infant and child immunization were not modified. The results of these studies were debated mainly because most of them had significant methodological limitations, enrolling a small number of unvaccinated subjects or being statistically underpowered. Moreover, these reports were counterbalanced by a large number of studies showing that vaccines were safe and not associated with an increased risk of T1D development, even when infectious agents included in the vaccines had been found to be associated with this disease. A protective effect was even evidenced in some studies (75). The findings of these studies can be illustrated by some examples. In a case-control study carried out in Sweden that included 339 cases and 528 controls (87), BCG, smallpox, pertussis, tetanus, rubella, and mumps vaccines had no influence on T1D epidemiology, whereas measles vaccine was associated with protection from T1D development (OR = 0.69; 95% CI 0.48-0.98). In a retrospective study carried out in Canada in which BCG vaccination was evaluated, a trend in favour of a protective effect of the vaccine was found, even though the small number of children receiving the BCG vaccine did not allow for drawing firm conclusions (88). Among children vaccinated at birth, only one (3.3%) was diagnosed with T1D by the age of 5 years, compared with 52 (24.5%) who had not been vaccinated (P < 0.01) (88). A 10-year follow-up study carried out in Finland, where a relationship between Hib vaccine and T1D development had been speculated a few years after introduction of the vaccine (83), did not implicate this vaccine regard and showed no significant difference in risk between children vaccinated against Hib at the age of 3 months and at the age of 24 months (89). A large, population-based, case-control study carried out in the USA reported that none of the evaluated vaccines was associated with an increased risk of T1D. The OR for the association with T1D was 0.28 (95% CI 0.07–1.06) for the whole cell pertussis vaccine, 1.36 (95% CI 0.70–2.63) for MMR, 1.14 (95% CI 0.51–2.57) for Hib, 0.81 (95% CI 0.52–1.27) for the HB vaccine, 1.16 (95% CI 0.72–1.89) for the varicella vaccine, and 0.92 (95% CI 0.53–1.57) for acellular pertussis-containing vaccines. Regarding the HB vaccine, it was shown that the vaccine was safe and that the risk of T1D did not differ between children at birth and those vaccinated later (90). A study in children with an increased genetic risk for T1D who received the influenza vaccine during the A/H1N1 2009 pandemic showed that this vaccine was not associated with an increased risk of islet autoimmunity, multiple islet autoantibodies or type 1. The hazard ratio [HR] (95% CI) for the appearance of at least one islet autoantibody was 0.75 (0.55-1.03), for at least two autoantibodies was 0.85 (0.57-1.26) and for T1D was 0.67 (0.42-1.07) (90). Regarding the HPV vaccine, no risk of T1D was found after HPV vaccine administration in two French studies (OR 1.2; 95% CI 0.4-3.6 in the first and HR 1.07; 95% CI 0.87-1.31 in the second) (91, 92). Similar results were reported in a retrospective cohort study carried out in the USA in which no increased risk of T1D associated with the HPV vaccine was found over the 10 years of the study period when comparing vaccinated with unvaccinated subjects (HR 1.21; 95% CI 0.94-1.57) (93). Moreover, autoimmune-specific safety analyses performed separately as part of this larger safety study noted a decreased association between HPV and new-onset T1D (HR 0.57; 95% CI 0.47-0.73) (94).

All these findings seem to indicate that the vaccines usually recommended for child protection are safe and not associated with the risk of T1D development, though it was not definitively established whether a certain degree of protection might be associated with very early administration of one or more vaccines. Some doubts may still exist for rotavirus vaccines. In general, the results of recently performed studies are conflicting, and a global evaluation of available data does not allow for firm conclusions, though the populations included in each database are quite different, as are the assumptions, inclusion criteria, and methods used for analysis. Moreover, it cannot be excluded that differences among studies are related to population variations in genetic background or other factors found to be associated with an increased risk of T1D development. Two studies carried out in Finland comparing the incidence of T1D in children with or without rotavirus vaccination showed no difference between the groups at short or long-time frames since immunization. The first study examined children at 4-6 years of age, and the absolute risk reduction of T1D development was 0.91 (95% CI 0.69–1.20) (95). In the second study enrolled children who had received the vaccine 11-14 years before, and the prevalence of T1D was similar in both groups, at 0.97% (25 of 2,580 children) in the control group and 1.04% (33 of 3,184 children) in the vaccine group (P = 0.810) (96). Conversely, completely different results have been reported by other studies. In studies carried out in Australia (97) and in the USA (98), vaccines were found to exert a protective effect, as the incidence of T1D measured before and after vaccine introduction decreased by 15% (relative risk [RR] 0.86; 95% CI 0.74-0.99) and 33% (HR 0.67; 95% CI 0.54-0.83), respectively, in vaccinated children. However, in the USA, differences between vaccines were attributed to a stronger effect of the pentavalent vaccine compared to the monovalent vaccine. Moreover, two very recent studies in which several sensitivity analyses to reduce the risk of bias were carried out did not find any influence of rotavirus vaccines on the risk of T1D (99, 100).

## Conclusions

T1D is not a rare disease. Nevertheless, several problems related to the use of vaccines in children with this disease have not been completely resolved, making administration of vaccines a challenge. T1D is considered a risk factor for infection development, and based on the incidence of infections in adults, mainly those with T2D, it is presumed that children are also at an increased risk of infection. However, data in this regard are scant, and the infections that must be monitored in children have not been established. To reduce the risk of infection, vaccines are strongly recommended in children with T1D. However, there are few studies regarding the immunogenicity and efficacy of vaccines in T1D children, and the need for different immunization schedules has not been precisely established. Fortunately, the previous presumed relationship between vaccine administration and T1D development appears to have been debunked, though some doubts regarding rotavirus vaccines remain. Further studies are needed to completely resolve the problems related to vaccine administration in T1D patients. In the meantime, the use of vaccines remains extensively recommended in children with this disease.

## Author Contributions

SE designed the project, co-wrote the first draft of the manuscript and supervised the activities. EMZ, LT, and SS participated in the preparation of the manuscript and literature review. MP and VP performed the literature review. BP and LI gave a substantial scientific contribution. NP co-wrote the first draft, revised the manuscript and made substantial scientific contributions. All authors contributed to the article and approved the submitted version.

## Funding

This review was supported by the Department of Medicine and Surgery, University of Parma, Parma, Italy.

## Conflict of Interest

The authors declare that the research was conducted in the absence of any commercial or financial relationships that could be construed as a potential conflict of interest.

## Publisher’s Note

All claims expressed in this article are solely those of the authors and do not necessarily represent those of their affiliated organizations, or those of the publisher, the editors and the reviewers. Any product that may be evaluated in this article, or claim that may be made by its manufacturer, is not guaranteed or endorsed by the publisher.
